# Author Correction: Synthetic RNA-based logic computation in mammalian cells

**DOI:** 10.1038/s41467-019-09906-3

**Published:** 2019-04-26

**Authors:** Satoshi Matsuura, Hiroki Ono, Shunsuke Kawasaki, Yi Kuang, Yoshihiko Fujita, Hirohide Saito

**Affiliations:** 1 Department of Life Science Frontiers, Center for iPS Cell Research and Application (CiRA), Kyoto University, Kyoto, 606-8507 Japan; 2 Graduate School of Medicine, Kyoto University, Kyoto, 606-8501 Japan; 3 Present Address: Department of Chemical and Biological Engineering, Hong Kong University of Science and Technology, Clear Water Bay, Hong Kong, SAR China

**Keywords:** RNA, Synthetic biology, Synthetic biology, miRNAs

Correction to: *Nature Communications* 10.1038/s41467-018-07181-2, published online 19 November 2018

The original version of this Article contained an error in the fourth sentence of the second paragraph of the ‘Improving the performance of miRNA-responsive circuits’ section of the Results, which incorrectly read ‘We confirmed a significant fold-change between ON and OFF states (from 3.5- to 9.0-fold) in 293FT cells (Supplementary Figure 3).’ The correct version states ‘4.6’ in place of ‘3.5’. This has been corrected in both the PDF and HTML versions of the Article.

The original version of the Supplementary Information contained a corresponding error in Supplementary Figure 3. The correct version of Supplementary Figure 3 is:

which replaces the previous incorrect version:

The HTML has been updated to include a corrected version of the Supplementary Information.

