# Supplementary material for: Synthetic RNA-based logic computation in mammalian cells
Source: Nat Commun. 2018 Nov 19;9:4847. doi: 10.1038/s41467-018-07181-2 (PMC6242901; doi:10.1038/s41467-018-07181-2)
Supplement: Supplementary file 1 — Supplementary Information [file 41467_2018_7181_MOESM1_ESM.pdf]

Supplementary Information

Synthetic RNA-based logic computation in mammalian cells

Matsuura et al.

This file includes:

Supplementary Figures 1 to 7

Supplementary Tables 1 to 5

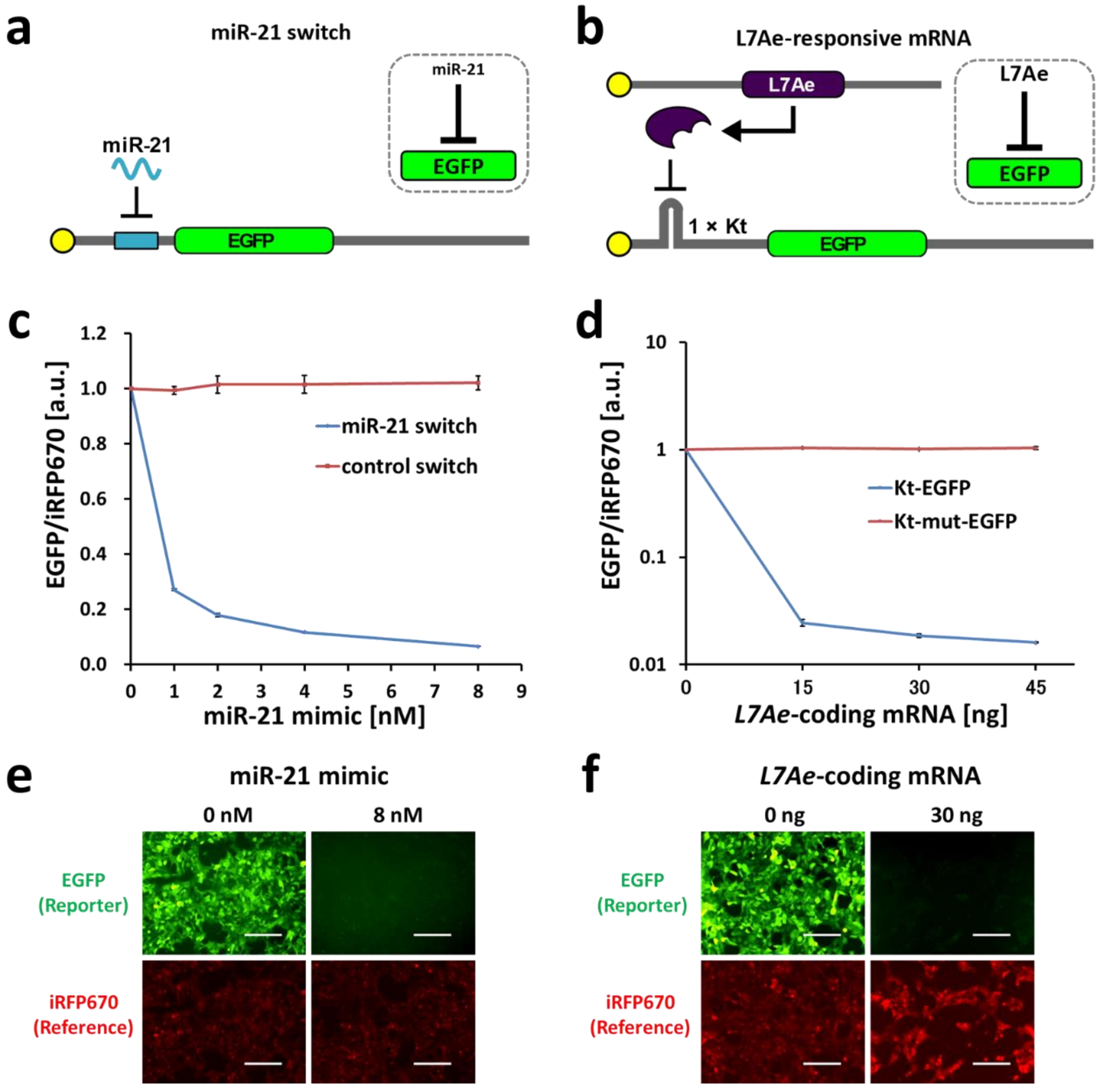

Supplementary Figure 1.

Characterization of miRNA switch and L7Ae-responsive mRNA.

(a, b) Schematic representation of the miR-21 switch (a) and the L7Ae-responsive mRNA (b). (c) Dose-response curves of the miR-21 switch with miR-21 mimic ranging from 0 to 8 nM in 293FT cells. Control switch is a mRNA without miRNA target sites. The highest fold-change was 15.3-fold, which was calculated by dividing the EGFP/iRFP670 ratio at 0 nM by that at 8 nM. (d) Dose-response curves of the L7Ae-responsive mRNA with *L7Ae*-coding mRNA ranging from 0 to 45 ng in 293FT cells. Kt-mut-EGFP mRNA is a mRNA with a mutant Kt motif within the 5'-UTR. Y-axis represents the mean EGFP per mean iRFP670 signal (EGFP/iRFP670). The ratio was normalized to the switch and the mRNA without miR-21 mimic (c) or *L7Ae*-coding mRNA (d), respectively. Data are shown as the mean ± s.d. (n = 3). (e) Representative microscopy images of cells co-transfected with the miRNA switch,

iRFP670-coding mRNAs and miR-21 mimic (0 nM or 8 nM). (f) Representative microscopy images of cells co-transfected with the L7Ae-responsive mRNA, *iRFP670*-coding mRNAs and *L7Ae*-coding mRNAs (0 ng or 30 ng). EGFP and iRFP670 were used as the reporter and the reference, respectively. Scale bars indicate 200 μm. Transfection details are described in Supplementary Table 4.

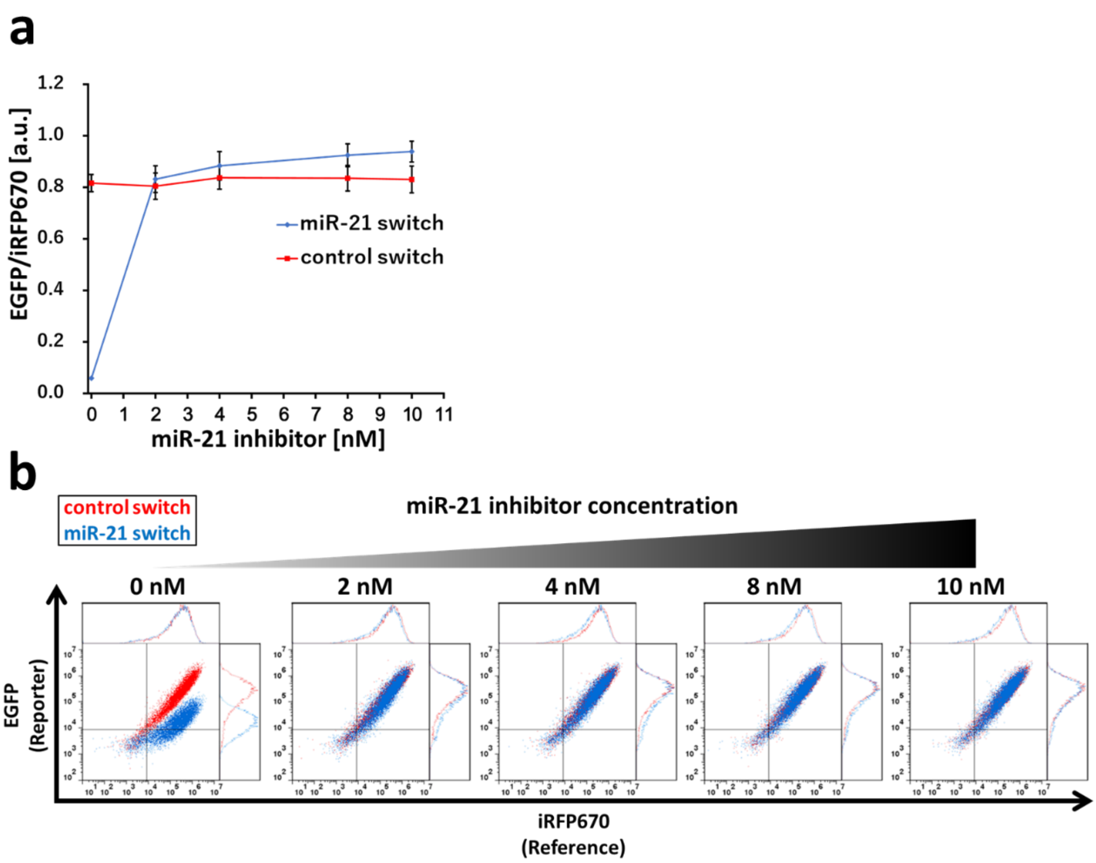

Supplementary Figure 2.

miRNA switch in HeLa cells.

Co-transfection of miR-21 switch and miR-21 inhibitor into HeLa cells. (a) Dose-response curves of the miR-21 switch with the miR-21 inhibitor ranging from 0 to 10 nM in HeLa cells. Control switch is a mRNA without miRNA target sites. Y-axis represents the mean EGFP per mean iRFP670 signal (EGFP/iRFP670). The ratios were not normalized. The highest fold-change was 15.8-fold, which was calculated by dividing the EGFP/iRFP670 ratio at 0 nM by that at 10 nM. Data are shown as the mean ± s.d. (n = 3). (b) Representative scatter plots and histograms for each switch in HeLa cells. Gates shown on the plots were generated by negative control (non-transfected) cells. Transfection details are described in Supplementary Table 4.

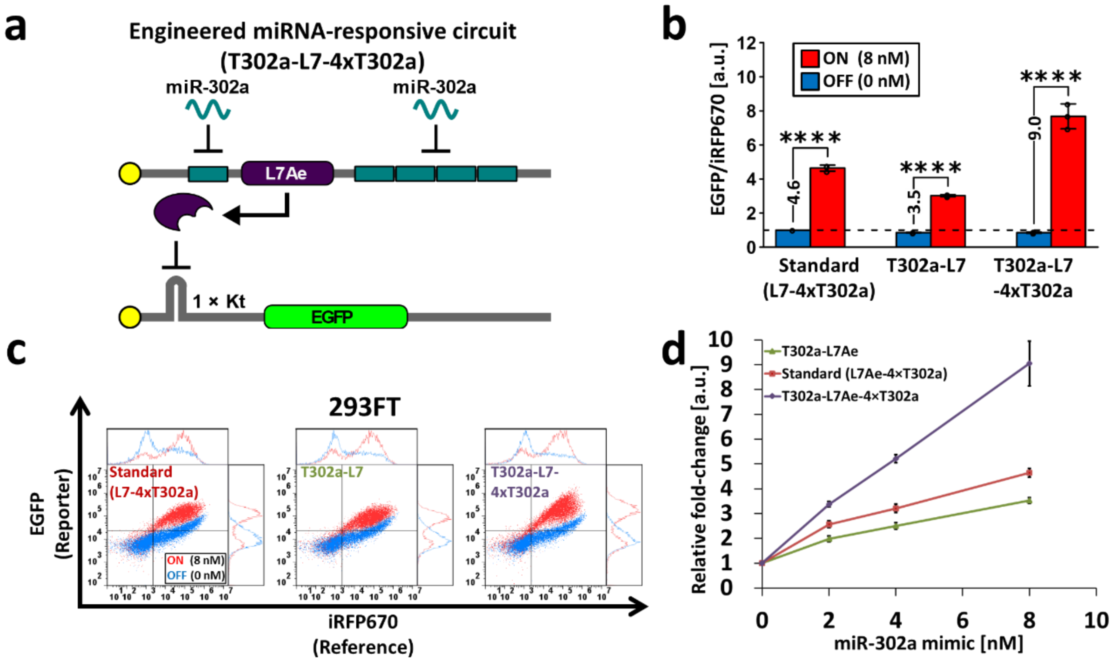

Supplementary Figure 3.

Engineering of L7Ae-mediated miR-302a circuits.

(a) Schematic representation of engineered L7Ae-mediated miR-302a-responsive circuits. (b) The bar charts represent the output level (EGFP/iRFP670) for each circuit in 293FT cells. The output levels in OFF states of standard circuits were normalized to 1. (c) Representative scatter plots and histograms for each circuit in 293FT cells. Gates shown on the plots were generated by negative control (non-transfected) cells. ON state and OFF state (red and blue) show the results in the presence and absence of input miRNAs, respectively. ON states in 293FT cells show cells co-transfected with each circuit and miR-302a mimic (8 nM). (d) Dose-response curves of the engineered L7Ae-mediated miRNA circuits with miR-302a mimic ranging from 0 to 8 nM in 293FT cells. The EGFP/iRFP670 ratio in each circuit without miR-302a mimic was set to 1. Data are shown as the mean ± s.d. (n = 3). The levels of significance (unpaired two-tailed Student's t-test) are denoted as ****P < 0.0001. Transfection details are described in Supplementary Table 4.

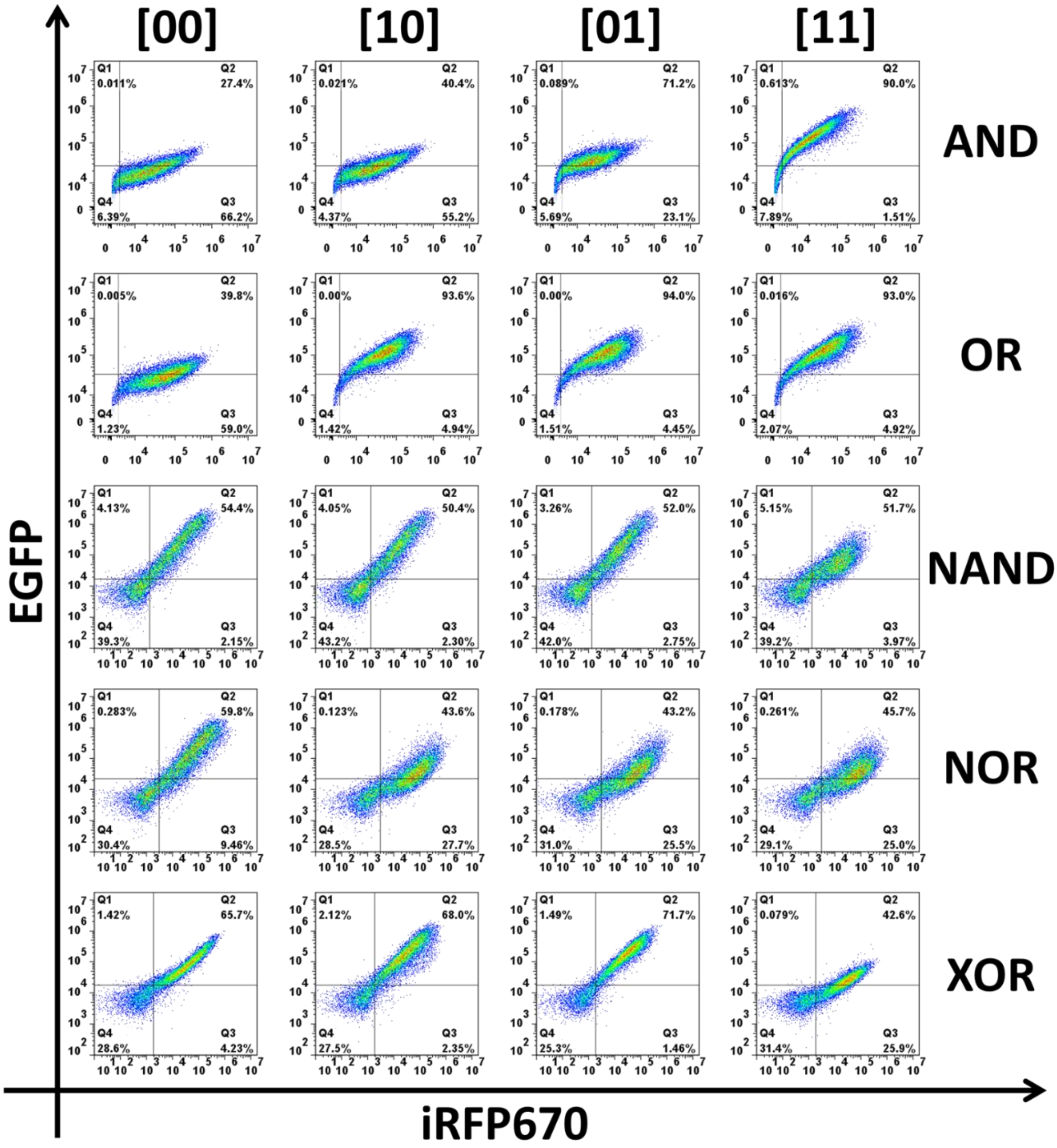

Supplementary Figure 4.

Representative flow cytometry data of the 2-input logic circuits shown in Fig. 3.

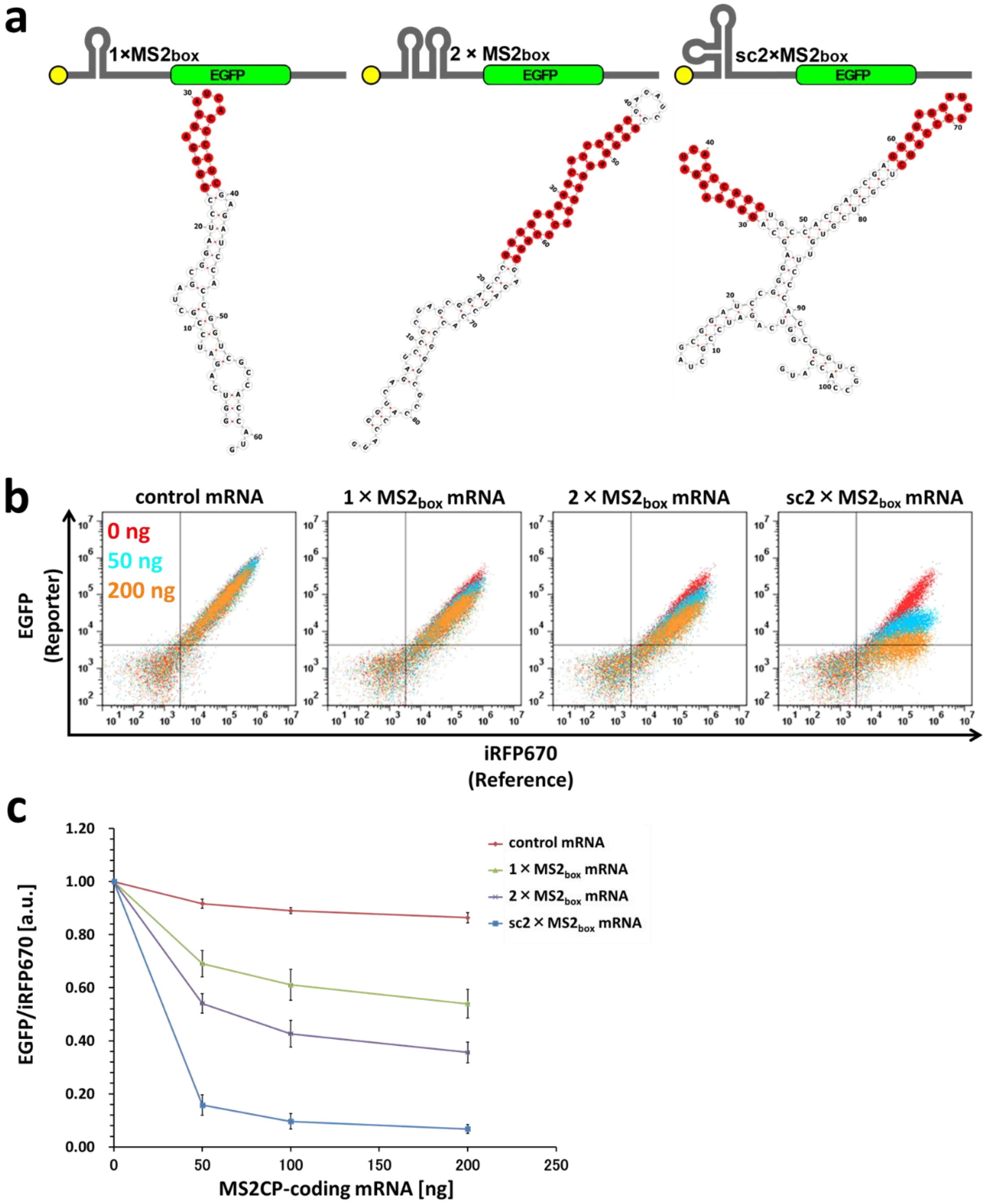

Supplementary Figure 5.

Engineering of MS2CP-responsive mRNAs.

We designed three kinds of MS2CP-responsive mRNAs. (a) Schematic representation of the MS2CP-responsive mRNAs (top) and the predicted minimum free energy structure of the 5'-UTR in each mRNA (bottom) by RNAfold (http://rna.tbi.univie.ac.at/cgi-bin/RNAWebSuite/RNAfold.cgi). The structures were visualized by forna

(http://rna.tbi.univie.ac.at/forna/). Red colored sequences mark the binding motifs ($MS2_{box}$). (b) Representative scatter plots for each MS2CP-resonsive mRNA with 0 ng, 50 ng and 200 ng *MS2CP*-coding mRNAs in 293FT cells. Gates shown on the plots were generated by negative control (non-transfected) cells. (c) Dose-response curves of the MS2CP-responsive mRNAs with control mRNA ranging from 0 to 200 ng in 293FT cells. Control mRNA is mRNA without the MS2CP binding motif within the 5'-UTR. Y-axis represents the mean EGFP per mean iRFP670 signal (EGFP/iRFP670). Ratios were normalized to each MS2CP-responsive mRNA without *MS2CP*-coding mRNA. Data are shown as the mean ± s.d. (n = 3). Transfection details are described in Supplementary Table 4.

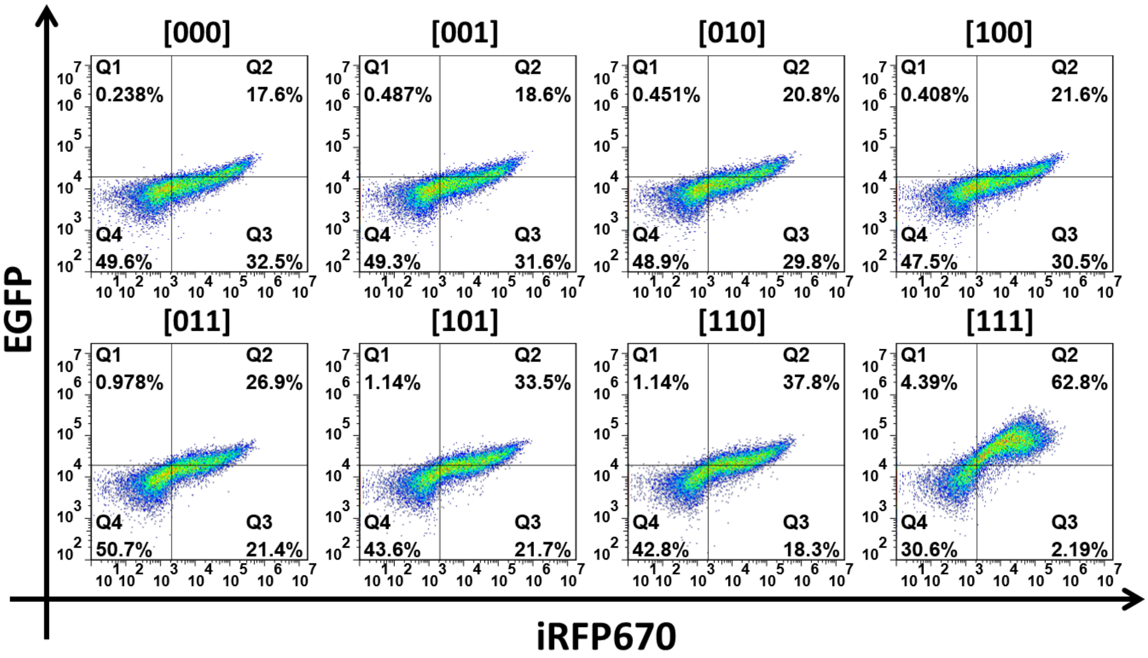

Supplementary Figure 6.

Representative flow cytometry data of the 3-input AND circuit shown in Fig. 4.

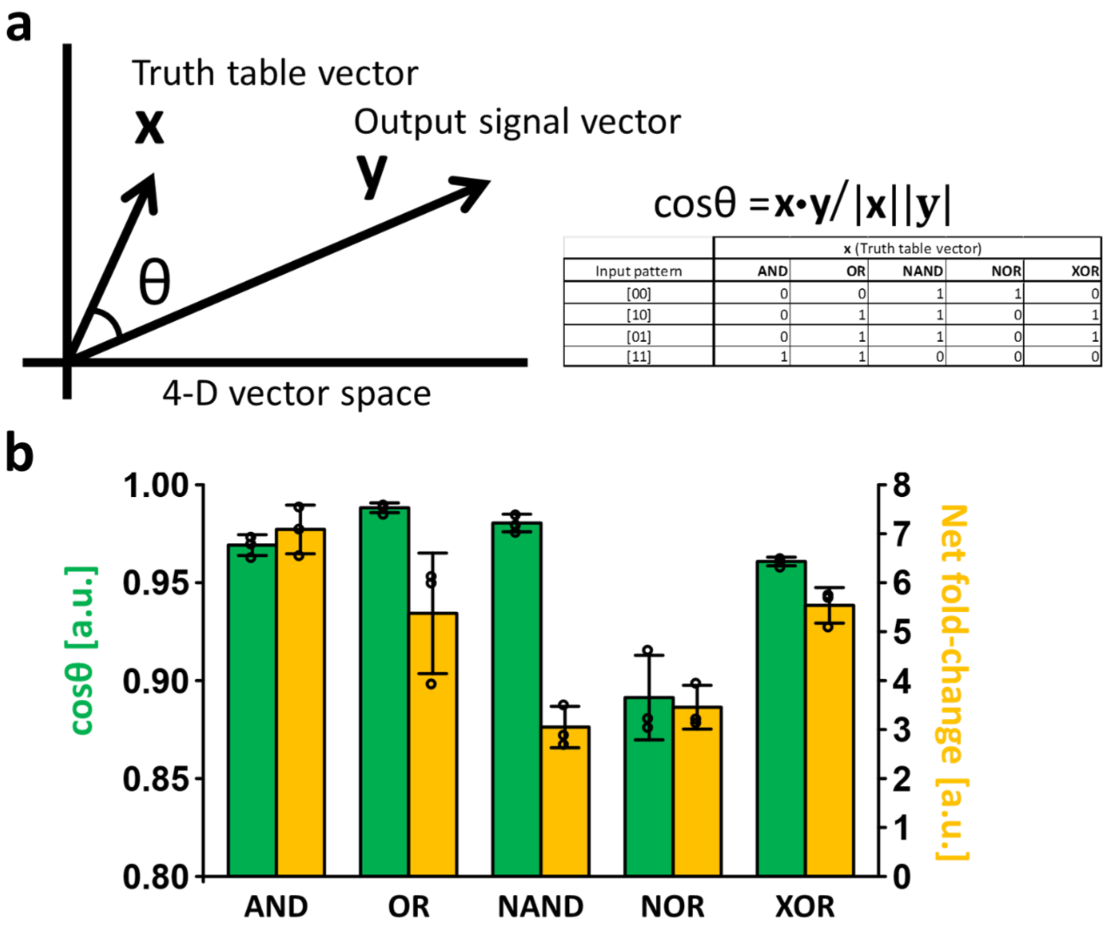

	x (Truth table vector)				
Input pattern	AND	OR	NAND	NOR	XOR
[00]	0	0	1	1	0
[10]	0	1	1	0	1
[01]	0	1	1	0	1
[11]	1	1	0	0	0

Supplementary Figure 7.

Performance validation of each logic circuit based on cosine similarity and net fold-change.

(a) Cosine similarity is calculated by the inner product between **x** (truth table vector) and **y** (output signal vector). (b) Validation of the performance of each logic circuit by cosine similarity and net fold-change. The results show the mean ± s.d. (n = 3).

Supplementary Table 1.

Tukey’s multiple comparisons test based on the experiments in Fig. 3 (2-input miRNA-responsive logic circuits). The levels of significance are denoted as *$P < 0.05$, **$P < 0.01$, ***$P < 0.001$, ****$P < 0.0001$, and n.s., not significant ($P \geq 0.05$).

Tukey's multiple comparisons test	AND	OR	NAND	NOR	XOR
[01]-[00]	n.s.	**	n.s.	****	****
[10]-[00]	n.s.	**	n.s.	****	****
[11]-[00]	****	**	***	****	**
[10]-[01]	n.s.	n.s.	n.s.	n.s.	*
[11]-[01]	****	n.s.	**	n.s.	****
[11]-[10]	****	n.s.	***	n.s.	****

Supplementary Table 2.

Tukey’s multiple comparisons test based on the experiments in Fig. 4 (3-input AND circuit). The levels of significance are denoted as ****P < 0.0001 and n.s., not significant ($P \geq 0.05$).

Tukey's multiple comparisons test			
3-input AND			
[001]-[000]	n.s.	[100]-[010]	n.s.
[010]-[000]	n.s.	[101]-[010]	n.s.
[011]-[000]	n.s.	[110]-[010]	n.s.
[100]-[000]	n.s.	[111]-[010]	****
[101]-[000]	n.s.	[100]-[011]	n.s.
[110]-[000]	n.s.	[101]-[011]	n.s.
[111]-[000]	****	[110]-[011]	n.s.
[010]-[001]	n.s.	[111]-[011]	****
[011]-[001]	n.s.	[101]-[100]	n.s.
[100]-[001]	n.s.	[110]-[100]	n.s.
[101]-[001]	n.s.	[111]-[100]	****
[110]-[001]	n.s.	[110]-[101]	n.s.
[111]-[001]	****	[111]-[101]	****
[011]-[010]	n.s.	[111]-[110]	****

Supplementary Table 3.

Tukey's multiple comparisons test based on the experiments in Fig. 5 (apoptosis regulatory 2-input AND circuit). The levels of significance are denoted as ****P < 0.0001 and n.s., not significant ($P \geq 0.05$).

Tukey's multiple comparisons test	apoptosis regulatory AND circuit	
	SYTOX red	Annexin V
[01]-[00]	n.s.	n.s.
[10]-[00]	n.s.	n.s.
[11]-[00]	****	****
[10]-[01]	n.s.	n.s.
[11]-[01]	****	****
[11]-[10]	****	****

Supplementary Table 4.

Transfection tables for all experiments in this study.

Figure 2b, c, d

Circuit	Standard (L7-4xT21)	T21-L7	T21-L7-4xT21
L7Ae-4×T21	30 ng		
T21-L7Ae		30 ng	
T21-L7Ae-4×T21			30 ng
Kt-EGFP	100 ng	100 ng	100 ng
iRFP670	100 ng	100 ng	100 ng
miR-21 mimic	0, 1, 2, 4 pmol		
miRNA mimic control	4, 3, 2, 0 pmol		
Stemfect	1 μL	1 μL	1 μL

Figure 2e, f

Circuit	Standard (L7-4xT21)	T21-L7-4xT21
L7Ae-4×T21	30 ng	
T21-L7Ae-4×T21		30 ng
Kt-EGFP	100 ng	100 ng
iRFP670	100 ng	100 ng
miR-21 inhibitor	0, 4 pmol	
miRNA inhibitor control	4, 0 pmol	
MessengerMax	1 μL	1 μL

Figure 3

Circuit	AND			
**Input pattern**	**[00]**	**[10]**	**[01]**	**[11]**
T21-L7Ae-4×T21	15 ng	15 ng	15 ng	15 ng
T302a-L7Ae-4×T302a	15 ng	15 ng	15 ng	15 ng
Kt-EGFP	100 ng	100 ng	100 ng	100 ng
iRFP670	100 ng	100 ng	100 ng	100 ng
miR-21 mimic		4 pmol		4 pmol
miR-302a mimic			4 pmol	4 pmol
miRNA mimic control	8 pmol	4 pmol	4 pmol	
Stemfect	1 μL	1 μL	1 μL	1 μL

Circuit	OR			
**Input pattern**	**[00]**	**[10]**	**[01]**	**[11]**
T21-T302a-L7Ae-4×T21	15 ng	15 ng	15 ng	15 ng
Kt-EGFP	100 ng	100 ng	100 ng	100 ng
iRFP670	100 ng	100 ng	100 ng	100 ng
miR-21 mimic		4 pmol		4 pmol
miR-302a mimic			4 pmol	4 pmol
miRNA mimic control	8 pmol	4 pmol	4 pmol	
Stemfect	1 μL	1 μL	1 μL	1 μL

Circuit	NAND			
**Input pattern**	**[00]**	**[10]**	**[01]**	**[11]**
T21-L7Ae-4×T21	15 ng	15 ng	15 ng	15 ng
T302a-L7Ae-4×T302a	15 ng	15 ng	15 ng	15 ng
Kt-MS2CP	150 ng	150 ng	150 ng	150 ng
2xMS2$_{box}$-EGFP (with m1pU, C)	200 ng	200 ng	200 ng	200 ng
iRFP670	100 ng	100 ng	100 ng	100 ng
miR-21 mimic		4 pmol		4 pmol
miR-302a mimic			4 pmol	4 pmol
miRNA mimic control	8 pmol	4 pmol	4 pmol	
MessengerMAX	1 μL	1 μL	1 μL	1 μL

**Circuit**	**NOR**			
**Input pattern**	**[00]**	**[10]**	**[01]**	**[11]**
T21-T302a-L7Ae-4×T21	15 ng	15 ng	15 ng	15 ng
Kt-MS2CP	100 ng	100 ng	100 ng	100 ng
2xMS2$_{box}$-EGFP (with m1pU, C)	100 ng	100 ng	100 ng	100 ng
iRFP670	100 ng	100 ng	100 ng	100 ng
miR-21 mimic		4 pmol		4 pmol
miR-302a mimic			4 pmol	4 pmol
miRNA mimic control	8 pmol	4 pmol	4 pmol	
MessengerMAX	1 µL	1 µL	1 µL	1 µL

**Circuit**	**XOR**			
**Input pattern**	**[00]**	**[10]**	**[01]**	**[11]**
T302a-L7Ae-4×T302a	15 ng	15 ng	15 ng	15 ng
T21-MS2CP-4×T21	100 ng	100 ng	100 ng	100 ng
2xMS2$_{box}$-T302a-EGFP-4×T302a (with m1pU, C)	50 ng	50 ng	50 ng	50 ng
Kt-T21-EGFP-4×T21	100 ng	100 ng	100 ng	100 ng
iRFP670	100 ng	100 ng	100 ng	100 ng
miR-21 mimic		4 pmol		4 pmol
miR-302a mimic			4 pmol	4 pmol
miRNA mimic control	8 pmol	4 pmol	4 pmol	
MessengerMAX	1 µL	1 µL	1 µL	1 µL

Figure 4b

Circuit	3-input AND							
**Input pattern**	**[000]**	**[001]**	**[010]**	**[100]**	**[011]**	**[101]**	**[110]**	**[111]**
T21-L7Ae-4×T21	15 ng	15 ng	15 ng	15 ng	15 ng	15 ng	15 ng	15 ng
T302a-L7Ae-4×T302a	15 ng	15 ng	15 ng	15 ng	15 ng	15 ng	15 ng	15 ng
T206-L7Ae-4×T206	15 ng	15 ng	15 ng	15 ng	15 ng	15 ng	15 ng	15 ng
Kt-EGFP	100 ng	100 ng	100 ng	100 ng	100 ng	100 ng	100 ng	100 ng
iRFP670	100 ng	100 ng	100 ng	100 ng	100 ng	100 ng	100 ng	100 ng
miR-21 mimic		4 pmol			4 pmol	4 pmol		4 pmol
miR-302a mimic			4 pmol		4 pmol		4 pmol	4 pmol
miR-206 mimic				4 pmol		4 pmol	4 pmol	4 pmol
miRNA mimic control	12 pmol	8 pmol	8 pmol	8 pmol	4 pmol	4 pmol	4 pmol	
MessengerMAX	1 µL	1 µL	1 µL	1 µL	1 µL	1 µL	1 µL	1 µL

Figure 5c, d

Circuit	Apoptosis regulatory 2-input AND				hBax only
**Input pattern**	**[00]**	**[10]**	**[01]**	**[11]**	
T206-L7Ae-P2A-Bcl2-4×T206	15 ng	15 ng	15 ng	15 ng	
T302a-L7Ae-P2A-Bcl2-4×T302a	15 ng	15 ng	15 ng	15 ng	
Kt-hBax	300 ng	300 ng	300 ng	300 ng	
hBax					300 ng
miR-206 mimic		2 pmol		2 pmol	
miR-302a mimic			2 pmol	2 pmol	
miRNA mimic control	4 pmol	2 pmol	2 pmol		
MessengerMAX	1 µL	1 µL	1 µL	1 µL	1 µL

Supplementary Figure 1c

switch	miR-21 switch	Control switch (EGFP)
T21-EGFP	100 ng	
EGFP		100 ng
iRFP670	100 ng	100 ng
miR-21 mimic	0, 0.5, 1, 2, 4 pmol	0, 0.5, 1, 2, 4 pmol
miRNA mimic control	4, 3.5, 3, 2, 0 pmol	4, 3.5, 3, 2, 0 pmol
MessengerMAX	1 μL	1 μL

Supplementary Figure 1d

Protein-responsive mRNA	L7Ae-responsive mRNA (Kt-EGFP)	Control mRNA (Kt-mut-EGFP)
L7Ae	0, 15, 30, 45 ng	0, 15, 30, 45 ng
Kt-EGFP	100 ng	
Kt-mut-EGFP		100 ng
iRFP670	100 ng	100 ng
MessengerMAX	1 μL	1 μL

Supplementary Figure 2a, b

switch	miR-21 switch	Control switch (EGFP)
T21-EGFP	100 ng	
EGFP		100 ng
iRFP670	100 ng	100 ng
miR-21 inhibitor	0, 1, 2, 4, 5 pmol	0, 1, 2, 4, 5 pmol
miRNA inhibitor control	5, 4, 3, 1, 0 pmol	5, 4, 3, 1, 0 pmol
MessengerMAX	1 μL	1 μL

Supplementary Figure 3b, c, d

**Circuit**	**Standard (L7-4xT302a)**	**T302a-L7**	**T302a-L7-4xT302a**
L7Ae-4×T302a	30 ng		
T302a-L7Ae		30 ng	
T302a-L7Ae-4×T302a			30 ng
Kt-EGFP	100 ng	100 ng	100 ng
iRFP670	100 ng	100 ng	100 ng
miR-302a mimic	0, 1, 2, 4 pmol		
miRNA mimic control	4, 3, 2, 0 pmol		
MessengerMAX	1 µL	1 µL	1 µL

Supplementary Figure 5b

**Protein-responsive mRNA**	**MS2CP-responsive mRNA (sc2xMS2$_{box}$)**	**MS2CP-responsive mRNA (2xMS2$_{box}$)**
MS2CP	0, 50, 100, 200 ng	0, 50, 100, 200 ng
sc2xMS2$_{box}$-EGFP	100 ng	
2xMS2$_{box}$-EGFP		100 ng
iRFP670	100 ng	100 ng
MessengerMAX	1 µL	1 µL
**Protein-responsive mRNA**	**MS2CP-responsive mRNA (1xMS2$_{box}$)**	**control mRNA (EGFP)**
MS2CP	0, 50, 100, 200 ng	0, 50, 100, 200 ng
1xMS2$_{box}$-EGFP	100 ng	
EGFP		100 ng
iRFP670	100 ng	100 ng
MessengerMAX	1 µL	1 µL

Supplementary Table 5.

List of primers and oligonucleotides used in this study.

**Fwd and Rev Primer**		
Name	*Type*	*Sequence (5'>3')*
TAP_T7_G3C fwd primer	5'-UTR	CAGTGAATTGTAATACGACTCACTATAGGGC
Rev5UTR	5'-UTR	CATGGTGGCGACCGGTGTCTTATATTTCTTCTTACTC
GCT7pro_5UTR2	5'-UTR	GCTAATACGACTCACTATAGGTTCCTTAATCGCGGATCC
L7Ae_IVTfwd	ORF	CACCGGTCGCCACCATGTACGTGAGATTTGAGGTTCCTG
TAP_IVTrev	ORF	GCCCCGCAGAAGGTCTAGACTATCACTCGAGATGCATATGAGATC
Fwd3UTR	3'-UTR	TCTAGACCTTCTGCGGGGC
Rev3UTR2T20	3'UTR	TTTTTTTTTTTTTTTTTTTTCCTACTCAGGCTTTATTCAAAGACCAAG
3UTR120A	3'-UTR	TTTTTTTTTTTTTTTTTTTTTTTTTTTTTTTTTTTTTTTTTTTTTTTTTTTTTTTTTTTTTTTTTTTTTTTTTTTTTTTTTTTTTTTTTTTTTTTTTTTTTTTTTTTTTTTTTTTTTTCCTACTCAGGCTTTATTCA
Fwd3UTRmiR	3'-UTR	TCTAGACCTTCTGCGGGGCGACGAGCTGTACAAGTAATTCTAGAAGATC
Rev 3UTR miRT20	3'-UTR	TTTTTTTTTTTTTTTTTTTTCCTACTCAGGCTTTATTCACGCTTTACTTGTACGCCCG
KWC0131_T7-kt-EGFP_Fw	5'-UTR	GAAATTAATACGACTCACTATAGGATCCGTGATCGGAAACGTGAGATCCACCTCAGATCCGCTAGGACACCCGCAGATCG
TAPEGFP_IVTfwd	ORF	CACCGGTCGCCACCATGGGATCCGTGAGCAAGGGC
Rev3UTR for 4×T	3'-UTR	TTTTTTTTTTTTTTTTTTTTCCTACTCAGGCTTTATTCAC
TAP_MS2CP_IVTfwd	ORF	CACCGGTCGCCACCATGGGATCCGCTTC
GCT7CMV_G	5'-UTR	GCTAATACGACTCACTATAGGTCAGATCCGCTAGCG

Rev5UTR for MS2	5'-UTR	CATGGTGGCGACCGGTGGG
MS2-T302a_Rev5UTR	5'-UTR	CATGGTGGCGACCGGTGTGATGTACTTAAACGTGGATGTACTTGCTATTACCGGGAACACGAGCGAG
Rev3UTR for 4×T	3'-UTR	TTTTTTTTTTTTTTTTTTTTCCTACTCAGGCTTTATTCAC
hBax_IVTfwd	ORF	CACCGGTCGCCACCATGGACGGGTC
hBax_IVTrev	ORF	GCCCCGCAGAAGGTCTAGAGTCAGCCCATCTTCTTCCAGATG
5UTR_sc_rev	5'-UTR	CATGGTGGCGACCGGTGGGAAC
Rev_5UTRmot	5'-UTR	CATGGTGGCGACCGGTGGATC

**Oligonucleotide**		
Name	*Type*	*Sequence (5'>3')*
IVT_5prime_UTR	5'-UTR	CAGTGAATTGTAATACGACTCACTATAGGGCGAATTAAGAGAGAAAAGAAGAGTAAGAAGAAATATAAGACACCGGTCGCCACCATG
5UTRtemp_T21-5p	5'-UTR	CGACTCACTATAGGTTCCGCGATCGCGGATCCTCAACATCAGTCTGATAAGCTAAGATCACACCGGTCGCCACCATG
IVT_3prime_UTR	3'-UTR	TCTAGACCTTCTGCGGGGCTTGCCTTCTGGCCATGCCCTTCTTCTCTCCCTTGCACCTGTACCTCTTGGTCTTTGAATAAAGCCTGAGTAGG
Spacer5UTRFwd	5'-UTR	ACACCCGCAGATCGAGAAGAAGGCGAATTAAGAGAGAAAAGAAGAGTAAGAAGAAATATAAGACACCGGTCGCCACCATG
3UTRtemp_4×T302a-5p	3'-UTR	CCTACTCAGGCTTTATTCACGCGGCCGCACTTAAACGTGGATGTACTTGCTACTTAAACGTGGATGTACTTGCTACTTAAACGTGGATGTACTTGCTACTTAAACGTGGATGTACTTGCTCAGCTCGTCGCCCCGCAGAAGGTCTAGA
5UTRtemp_T21-5p-T302a-5p	5'-UTR	CGACTCACTATAGGTTCCGCGATCGCGGATCCTCAACATCAGTCTGATAAGCTAAGATAAAGCAAGTACATCCACGTTTAAGTCACCGGTCGCCACC

		ATG
Spacer+T21 5UTRFwd	5'-UTR	ACACCCGCAGATCGAGAAGAAGGCGAATTAAGAGATCAACATCAGTCTGATAAGCTAATAAGACACCGGTCGCCACCATG
3UTRtemp_4xT206	3'-UTR	CCTACTCAGGCTTTATTCACGCGGCCGCTGGAATGTAAGGAAGTGTGTGGTGGAATGTAAGGAAGTGTGTGGTGGAATGTAAGGAAGTGTGTGGTGGAATGTAAGGAAGTGTGTGGCAGCTCGTCGCCCCGCAGAAGGTCTAGA
5UTR_MS2x1	5'-UTR	GGTCAGATCCGCTAGCGGATCCGGTGAGGATCACCCATCGAGATCCACCGGTCGCCACCATG
5UTR_MS2x2	5'-UTR	GGTCAGATCCGCTAGCGGATCCGGTGAGGATCACCCATCGAGATCCGGTGAGGATCACCCATCGAGATCCACCGGTCGCCACCATG
